# Circadian and Light Regulated Expression of *CBF*s and their Upstream Signalling Genes in Barley

**DOI:** 10.3390/ijms18081828

**Published:** 2017-08-22

**Authors:** Krisztián Gierczik, Aliz Novák, Mohamed Ahres, András Székely, Alexandra Soltész, Ákos Boldizsár, Zsolt Gulyás, Balázs Kalapos, István Monostori, László Kozma-Bognár, Gábor Galiba, Attila Vágújfalvi

**Affiliations:** 1 Agricultural Institute, Centre for Agricultural Research, Hungarian Academy of Sciences, 2462 Martonvásár, Hungary; gierczik.krisztian@agrar.mta.hu (K.G.); novak.aliz@agrar.mta.hu (A.N.); ahres.mohamed@agrar.mta.hu (M.A.); szekely.andras@agrar.mta.hu (A.S.); soltesz.alexandra@agrar.mta.hu (A.S.); boldizsar.akos@agrar.mta.hu (Á.B.); gulyas.zsolt@agrar.mta.hu (Z.G.); kalapos.balazs@agrar.mta.hu (B.K.); monostori.istvan@agrar.mta.hu (I.M.); vagujfalvi.attila@agrar.mta.hu (A.V.); 2 Festetics Doctoral School, Georgikon Faculty, University of Pannonia, 8360 Keszthely, Hungary; 3 Institute of Plant Biology, Biological Research Centre, Hungarian Academy of Sciences, 6726 Szeged, Hungary; kozma_bognar.laszlo@brc.mta.hu; 4 Department of Genetics, Faculty of Sciences and Informatics, University of Szeged, 6726 Szeged, Hungary

**Keywords:** barley, circadian rhythm, gene expression, calcium signalling, HvCBF-phylogenetic subgroups, light quality, low red/far-red ratio

## Abstract

CBF (C-repeat binding factor) transcription factors show high expression levels in response to cold; moreover, they play a key regulatory role in cold acclimation processes. Recently, however, more and more information has led to the conclusion that, apart from cold, light—including its spectra—also has a crucial role in regulating *CBF* expression. Earlier, studies established that the expression patterns of some of these regulatory genes follow circadian rhythms. To understand more of this complex acclimation process, we studied the expression patterns of the signal transducing pathways, including signal perception, the circadian clock and phospholipid signalling pathways, upstream of the *CBF* gene regulatory hub. To exclude the confounding effect of cold, experiments were carried out at 22 °C. Our results show that the expression of genes implicated in the phospholipid signalling pathway follow a circadian rhythm. We demonstrated that, from among the tested *CBF* genes expressed in *Hordeum vulgare* (*Hv*) under our conditions, only the members of the HvCBF4-phylogenetic subgroup showed a circadian pattern. We found that the HvCBF4-subgroup genes were expressed late in the afternoon or early in the night. We also determined the expression changes under supplemental far-red illumination and established that the transcript accumulation had appeared four hours earlier and more intensely in several cases. Based on our results, we propose a model to illustrate the effect of the circadian clock and the quality of the light on the elements of signalling pathways upstream of the *HvCBF*s, thus integrating the complex regulation of the early cellular responses, which finally lead to an elevated abiotic stress tolerance.

## 1. Introduction

Plant cells perceive the external signals from the environment, transmit them intracellularly (signal transduction) and react with adequate responses. After stimulus detection, plant cells release messenger molecules. Among them is calcium ion (Ca^2+^) that plays a pivotal role in the signal transduction [1]. The amount of cytosolic calcium level ([Ca^2+^]_cyt._) is highly influenced by the phospholipid signalling pathway and is responsible for the calcium release from intracellular calcium stores, such as the vacuole or the endoplasmic reticulum [2,3].

Changes in the intracellular calcium ion levels are sensed by the Calcium Binding Proteins (CBPs) [4]. CBPs are classified into three groups: (1) calcineurin B-like proteins (CBLs); (2) calmodulin (CaM) and calmodulin-like proteins (CMLs); and (3) calcium-dependent protein kinases (CDPKs) and the calcium and calmodulin-dependent protein kinase (CCaMK) [1]. In *Arabidopsis thaliana*’s genome, about 250 encoded CBPs can be found [5]; this high number ensures adequate, fine-tuned responses to the many stimulus perceived by the cell. Binding of the calcium ion leads to conformational changes of the CBP, which in turn controls the expression of transcriptional regulators, such as C-repeat binding factors (CBFs) [6,7]. It has been reported that CaM-binding transcriptional activators (CAMTAs) bind to the *CBF2* gene promoter in *Arabidopsis*, thus proving the connection between the calcium signalling and the induction of CBF-regulated genes [8].

The CBFs, also known as dehydration-responsive element binding (DREB) factors [9], are transcription factors. They belong to the AP2/EREBP (APETALA2/ethylene-responsive element binding proteins) multigene family of DNA binding proteins [10]. The CBF/DREB (hereinafter CBF) proteins recognise a conserved RCCGAC core motif: the C-repeat (CRT) or dehydration-responsive elements (DRE) in the promoter of the target genes [11,12]. This CRT/DRE sequence is present in several drought or cold responsive genes, such as *AtRD29A* (responsive to desiccation), *HvDHN1*–*HvDHN11* (dehydrin), *AtKIN1* (cold-induced), *TaWCS120* (wheat cold-specific), *AtCOR15a* (cold-regulated) or *AtCOR6.6* [13,14,15].

The CBF regulatory network is one of the most important and well-described stress-related signalling systems in plants. *Arabidopsis* possesses three tandem repeats of *CBF* genes on chromosome 4: *CBF1*, *CBF2* and *CBF3*. These genes are rapidly induced by low temperature, which is followed by the increased expression of the CBF-regulated genes (CBF regulon), leading to an increased level of freezing tolerance [16,17]. On the other hand, the expression of the *CBF4* gene is up-regulated during drought or salt conditions but not under cold stress [18,19].

The barley (*Hordeum vulgare* L.) genome encodes at least 20 *HvCBF*s, which are divided into three phylogenetic groups: HvCBF1-, HvCBF3- and HvCBF4-subgroups [20]. All members of the HvCBF3- and HvCBF4-subgroups are localised on the long arm of chromosome 5, whereas the genes of the HvCBF1-subgroup are spread on other chromosomes [20,21]. Quantitative genetic studies in barley, in diploid einkorn wheat (*Triticum monococcum* L.) and in hexaploid wheat (*Triticum aestivum* L.) have shown that a large number of phenotypic differences in frost tolerance and winter hardiness are explained by two QTLs (Quantitative Trait Locus): by the *Fr-1* and *Fr-2* (*Frost resistance*) loci. Furthermore, at least 11 *CBF* genes have been mapped in the *Fr-2* locus within a small 0.7–0.8 centimorgan distance in diploid wheat and barley [21,22,23,24,25,26,27,28,29,30,31,32], clearly showing that the *CBF* genes are the best candidate genes for those QTLs.

In addition to low temperature, light also acts as an external signal which affects the expression level of the *CBF* genes [33]. It has been described that the photoperiod and the light quality are important regulators in the cold acclimation processes through the modulation of the CBF regulon [34,35,36]. In higher plants, photoreceptors are specific to different wavelengths that allow the detection of the duration, direction, quality and quantity of the light. Absorption of light at specific wavelengths initiates the corresponding signalling pathway [37]. The quality of light is perceived by phytochromes that absorb red (R; λ_max_ ~660 nm) and far-red (FR; λ_max_ ~730 nm), and by phototropins, cryptochromes, ZEITLUPE and UV-B receptors, which respond to blue and/or UV light [38,39,40].

Phytochromes exist in two basic forms: in the inactive R-absorbing P_r_ form and in the active FR-absorbing P_fr_ form. The P_r_ form is photoconverted into the P_fr_ form by exposure to red light. This process is reversible: it reverts rapidly back into the P_r_ form upon far-red illumination. The reversion from the active P_fr_ to the inactive P_r_ form occurs spontaneously, independent of the light conditions, by a slow-rate thermal relaxation called dark reversion [41]. An effective parameter to describe the natural light conditions is the ratio of R and FR photon irradiance (R/FR). The R/FR ratio is around the value of 1.37 (at latitude 42° N) at sunset, although 30 min after sunset it decreases to the value of 0.69 [42]. The decrease of R/FR ratio, i.e., during dusk, results in the conversion of the active P_fr_ form to the inactive P_r_ form. In *Arabidopsis* plants an increased expression of *CBF* genes was observed at 16 °C but not at 22 °C in response to low R/FR, suggesting that temperature and phytochrome signalling are closely related [43].

The expression level of the *CBF* genes peaks 8 h after dawn (zeitgeber time 8; ZT8) in long-day (LD, 16 h photoperiod) and short-day (SD, 8 h photoperiod) conditions as well, but the amplitude of the peaks are increased in the case of SD relative to LD [35]. This kind of rhythmicity in gene expression pattern is characterised as daily rhythm. Some of these rhythms are not directly driven by the light/dark conditions, but are governed by a self-sustaining endogenous oscillation with approximately 24 h periods and therefore could persist under constant conditions—a hallmark of circadian rhythms/oscillations.

This endogenous oscillation is ensured by the activity of the circadian clock, which was investigated mostly in *Arabidopsis*, as a model for plant molecular genetics. In the early morning, two closely related transcription factors, the *Late Elongated Hypocotyl* (*LHY*) and the *Circadian Clock Associated 1* (*CCA1*), are co-expressed. The transcription of these genes is repressed by the Pseudo-Response Regulator proteins (PRR7, PRR9 and especially by PRR1—also called Timing of CAB Expression 1 (TOC1)) during the afternoon and night as well. The repressed state of *LHY* and *CCA1* genes is released by the “evening-complex” (EC), a complex of three proteins: the Early Flowering 3 and 4 (ELF3 and ELF4) and the Lux Arrhythmo (LUX) proteins. The EC down regulates the *PRR7* and *PRR9* genes, thus de-repressing the transcription of *LHY* and *CCA1* genes in the morning [37,44,45]. The circadian clock controls the expression of a wide range of genes influencing all aspects of the plant life. Since it affects many cellular processes, such as signalling and metabolic pathways [46], it finally modulates many “basic” physiological functions, such as seed germination processes, gas exchange, photosynthetic activity or the regulation of flowering [47].

It has been long known that many changes in the environment lead to sudden bursts of Ca^2+^ in the cytosol, and also that Ca^2+^ is one of the most common secondary messengers. However, the cellular response is a consequence of many stimuli most of the time. Such is stomatal opening, which is regulated both by the [Ca^2+^]_cyt._ level and by the circadian clock as well [48]. Decreasing the concentration of the phosphatidylinositol 3-phosphate (PI3P) and phosphatidylinositol 4-phosphate (PI4P), which are members of the phospholipid signalling pathway, by blocking the phosphatidylinositol (PI) kinase activity (PI 3-kinase and PI 4-kinase) or by overexpressing PI3P- and PI4P-binding proteins, reduced the number of open stomata in a time of the day-specific manner, indicating that the clock could regulate stomatal opening via the modulation of Ca^2+^ signalling [47].

The first element of the phospholipid signalling pathway is the phosphatidylinositol transfer protein (PITP). The PITP transports the PI molecule between membrane bilayers, whereas the PI 4-kinase (PI4K) catalyses the phosphorylation of the PI molecule [3]. The production of PI4P, the only known precursor of PI 4,5-bisphosphate (PIP_2_) is catalysed by the PI 4-kinase (PI4K). PIP_2_ can be cleft by the membrane-bound phospholipase C (PLC) enzyme [49], catalysing the hydrolysis of the PIP_2_ and thus forming diacylglycerol (DAG) and inositol 1,4,5-triphosphate (IP_3_). Elevated IP_3_ level then activates the IP_3_ receptors on the endoplasmic reticulum membrane, which leads to calcium release into the cytoplasm [4,50]. The diacylglycerol kinase converts the DAG to phosphatidic acid (PA), a typical plant second messenger playing crucial roles in many abiotic and biotic stress signalling processes. PA can be generated either indirectly by PLC or directly via the phospholipase D (PLD) pathway, where PLD catalyses the hydrolysis of phospholipids like phosphatidylcholine to produce choline and PA [3,51].

Low temperature induces the expression of *CBF* genes, resulting in an increased level of frost tolerance. Our current knowledge is limited on those signalling pathways and their regulation, which lead to the induction of these genes. However, it has already been described in *Arabidopsis* that, besides temperature, the quality of light (low R/FR ratio) and the circadian clock are also modulators of *CBF* expression. One of the possibilities to study the regulation of the signalling elements would be the use of mutant lines. However, working with mutant cereal lines, such as barley, mutant for each element would be challenging, so, to get a first hint on the subject, we started our work by analysing the gene expression patterns of some representative components of the circadian clock and light regulatory signalling pathways. Thus, our aim was to find out whether the circadian clock and light—as signals—have the same modulatory effect on the signal transduction pathway in a model cereal organism, in barley.

## 2. Results

A winter habit barley variety, Nure, was grown with 12 h photoperiod in plant growth chambers. After the preliminary growth, the plantlets were exposed to modulated and unmodulated white light. In the case of modulated white light, supplemental FR light was added to reach low R/FR ratio. To determine the gene expression profiles, leaf samples were collected every 4 h during two days of light/dark cycles and two days in constant light. Gene expression was monitored with quantitative Real-Time PCR (qRT-PCR) method. The sequence identifiers of all the genes studied herein are listed in Table S1. In our recent works [36,52], *cyclophilin* proved to be a reliable reference gene in barley. Besides, in this current study, we also made sure that the expression of *cyclophilin* gene was not changed either in white light treated or in supplemental FR light treated samples. In the white light treated samples the detected *C*_t_ mean was 21.13 ± 0.82, while in the supplemental FR light treated samples the *C*_t_ mean was 21.68 ± 0.57. These values were originated from the three technical replicates of all the detected *C*_t_ values. Concerning these results we considered this gene as a reliable reference gene, and used it for normalisation. The mean Δ*C*_t_ values and the standard deviation data of the three technical replicates are presented in Table S2.

### 2.1. Expression Analysis of the Core Circadian Clock Genes

**Figure 1:**
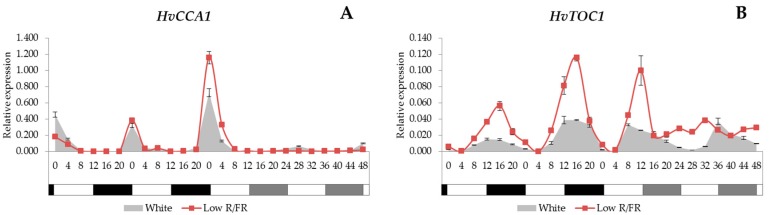
The expression patterns of the *HvCCA1* and *HvTOC1* (**A**,**B**) with white light and low R/FR illumination. In the first two days, the plants were illuminated for 12 h, whereas, in the next two days, they were kept under constant light conditions. Samples were collected every 4 h during four days. Transcript levels were calculated with the Δ*C*_t_ method. The values on the X-axes show the time in hours after dawn. The white and black bars symbolise the light and dark periods, while grey bars indicate subjective night. The data and error bars, which represent the standard deviation, originated from three technical replicates.

The analysis revealed that the expression of *HvCCA1* gene was induced intensively at dawn, and it showed a rhythmic pattern (Figure 1A). Under constant light conditions, the transcripts of *HvCCA1* gene were barely detected. With the supplemental FR illumination the expression level did not change dramatically. The transcription of the *HvTOC1* peaked early in the night; moreover, this gene kept its period and amplitudes under constant light conditions, showing circadian rhythm (Figure 1B). The low R/FR illumination triggered a higher level of gene expression in the case of normal light/dark cycles without affecting the phase. In low R/FR conditions, the amplitude of the *HvTOC1* gene was severely reduced under constant light, and a constant transcript level was observed.

### 2.2. Expression Analysis of the Phospholipid Signalling Pathway Genes

**Figure 2:**
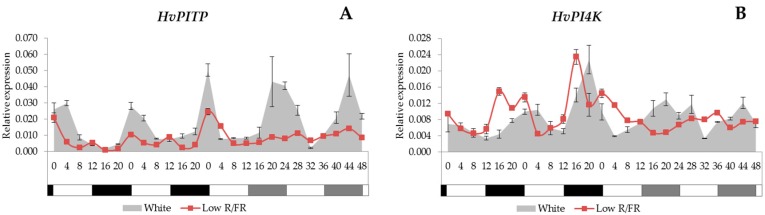
The expression patterns of *HvPITP* and *HvPI4K* genes (**A**,**B**) with white light and low R/FR illumination. Conditions are the same as in Figure 1.

We examined the gene expression patterns of the *PITP* and the *PI4K* from the phospholipid signalling pathway. We found that the *HvPITP* gene was controlled by the circadian clock and it showed a morning-phased expression (Figure 2A). The supplemental FR illumination decreased the transcript level of the *HvPITP* gene, and rhythmicity under constant conditions was lost.

The *HvPI4K* expression analysis revealed that this gene was transcribed most intensively late in the night or early in the morning, and this periodicity was kept under constant conditions as well (Figure 2B). In the case of low R/FR conditions, *HvPI4K* expression displayed an early phase of expression under light/dark cycles, but the rhythmicity was lost under constant conditions.

### 2.3. Gene Expression Patterns of the Calcium Signalling Elements

In this study, we examined the gene expression patterns of a putative *PLC* gene (*HvPLC.1 (put.)*), a predicted *PLC* gene (*HvPLC (pred.)*) and a putative *PLD* gene (*HvPLD (put.)*) gene. We also analysed expression patterns of genes from each CBP family: a calcineurin B like protein (*HvCBL2*), two calmodulins (*HvCaM.1* and *HvCaM.2*) and a calcium dependent protein kinase (*HvCDPK12*). Since it had been demonstrated that CAMTAs present a link between calcium signalling and the cold induction of the CBF pathway [8], in this work, we determined the expression pattern of a predicted *CaM-binding transcriptional activator* gene (*HvCAMTA3 (pred.)*) as well.

**Figure 3:**
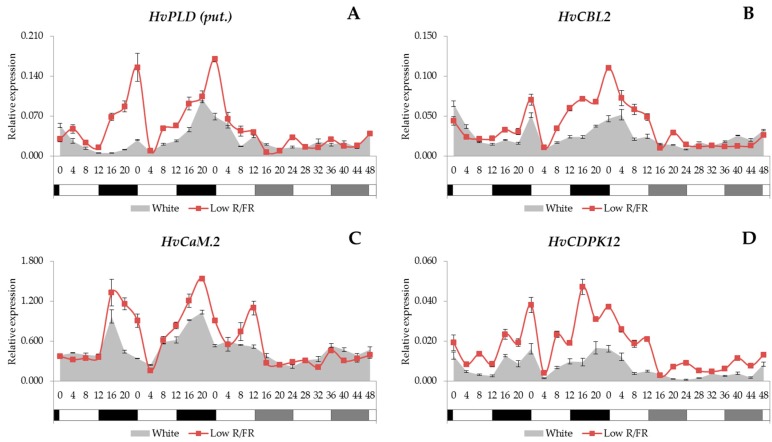
The expression patterns of the *HvPLD (put.)*, *HvCBL2*, *HvCaM.2*, *HvCDPK12* (**A**–**D**) with white light and low R/FR illumination. Conditions are the same as in Figure 1.

We found no common expression patterns that might be expected for all the genes related to the calcium signalling pathway, since the analysed genes showed three different expression patterns. In the case of *HvPLD (put.)*, *HvCBL2*, *HvCaM.2* and *HvCDPK12* genes (Figure 3A–D), we observed that the transcription was driven by the light/dark cycles and showed a peak at the end of the night. However, rhythmicity was lost under constant white light conditions. Supplemental FR light had no significant effect on the temporal pattern of expression under day/night cycles, but maximal transcript levels were increased in every case.

The transcription of *HvPLC (pred.)* and *HvCaM.1* genes showed no rhythmic patterns and was unaffected by low R/FR conditions (Figure S1A,B). We could not detect consistent daily/circadian rhythm in the expression patterns of *HvPLC.1 (put.)* and *HvCAMTA3 (pred.)* (Figure S2A,B), but under constant white light condition the transcript accumulation was reduced, and low R/FR induced the expression of these genes in light/dark cycles.

### 2.4. Expression Analysis of the HvCBF Genes

**Figure 4:**
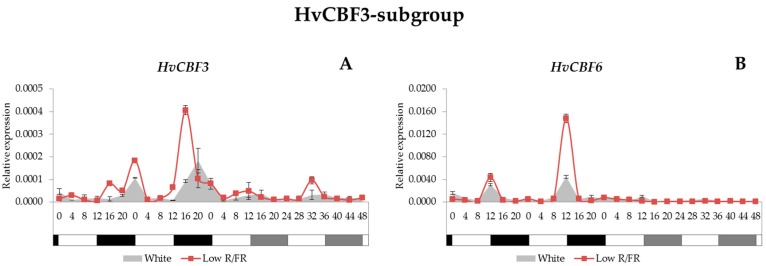
Gene expression patterns of the *HvCBF3* and *HvCBF6* (**A**,**B**) with white light and low R/FR ratio in the spectra. Conditions are the same as in Figure 1.

The barley genome contains at least 20 *CBF* genes that, based on phylogenetic analysis, are classified into three subgroups: HvCBF1, HvCBF3 and HvCBF4 [20]. For their characterisation we studied the expression patterns of the representative members from each subgroup. The HvCBF1-subgroup contains four *HvCBF* genes and two of them (*HvCBF1* and *HvCBF11*) were studied; however, the plant growth temperature (22 °C) was not inductive for these genes since we could not detect any transcripts. In the case of the HvCBF3-subgroup, we examined the expression levels of *HvCBF3*, -*6*, -*10A*, -*12*, and -*15* as well as *HvCBF16* genes. From these, only the *HvCBF3* and the *HvCBF6* were expressed in winter barley variety Nure at 22 °C. Concerning the expression patterns, we found that the *HvCBF3* gene was expressed at a very low level under light/dark cycles; even so, the transcription was detected late in the night and early in the morning (Figure 4A). Under constant white light condition, the expression level of this gene decreased even further. With supplemental FR illumination, the *HvCBF3* gene was expressed a few hours earlier compared with unmodulated white light condition, and showed elevated transcript levels under light/dark cycles. The expression of the *HvCBF6* gene showed a sharp peak consistently at the beginning of the night (Figure 4B). With supplemental FR light, the transcription level of this gene was elevated but without affecting the phase of expression. Expression of *HvCBF6* ceased under constant white light condition and constant low R/FR illumination as well.

**Figure 5:**
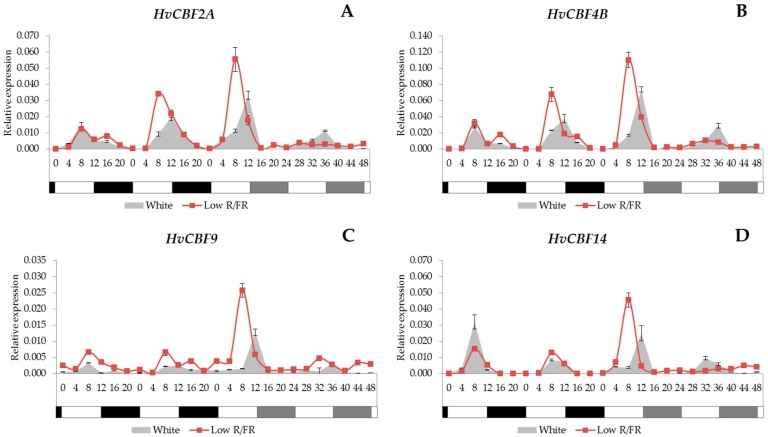
Gene expression levels in *HvCBF2A*, *HvCBF4B*, *HvCBF9* and *HvCBF14* (**A**–**D**) with white light and low R/FR illumination. Conditions are the same as in Figure 1.

Concerning the HvCBF4-subgroup, we examined the transcript levels of the *HvCBF2A*, -*4B*, and -*9* as well as *HvCBF14* genes. It was observed that all of them showed a significant similarity in their expression patterns (Figure 5A–D). We found that the peak of these four genes occurred 8–12 h after dawn. This rhythmicity was maintained under constant white light conditions as well. Low R/FR light advanced the phase and increased the amplitude of expression under light/dark cycles, but reduced overall expression levels under constant conditions; moreover, the circadian rhythm was lost with supplemental FR light treatment without a dark period.

## 3. Discussion

### 3.1. Clock Genes

The circadian clock in plants consists of the morning-phased and the evening-phased regulatory feedback loops interlocked by a central loop. One of the core components of the morning loop is the *CCA1* gene, while the *TOC1* gene is the member of the evening loop [53]. Surprisingly, in our experiment, the expression of *HvCCA1* was hardly detected under constant light, whereas the amplitude of *HvTOC1* expression was rapidly dampening in low R/FR conditions. Clock regulated output genes, such as *HvPITP*, showed robust rhythmicity under the same conditions, clearly demonstrating the proper operation of the clock mechanism. This might indicate that high level transcription of *HvCCA1* is not a prerequisite of the oscillator function. In seedlings, grown in constant light or dark conditions, both *HvCCA1* and *HvTOC1* are expressed without oscillations, demonstrating the importance of entrainment for clock functions [54]. Interestingly, *HvCCA1* showed a flat and very low level of expression in constant light, but its transcription was gradually increasing in constant darkness [54]. This could indicate that *HvCCA1* is repressed by light, thus explaining our results. Authors of another study monitored *HvCCA1* and *HvTOC1* expression in seedlings grown in constant darkness but entrained by temperature cycles [55]. The robust and rhythmic expression of *HvCCA1* under these conditions also supports the hypothesis that prolonged light treatments may reduce the transcription of *HvCCA1*.

In a recent study, the expression patterns of the *CCA1* and *TOC1* genes in *Arabidopsis* showed high degree of similarity to the ones that we observed in barley [56]. After free-running in constant light for one week, it was found in *Arabidopsis* that the *TOC1* gene kept its circadian rhythm with decreased peaks, while the *CCA1* gene lost its amplitude completely. The same tendency was recorded in our experiments (Figure 1A,B) carried out on a monocot species.

### 3.2. Ca^2+^ Signalling

In this study, we presented that the *PITP* and *PI4K* genes are controlled by the circadian clock. The *PITP* and *PI4K* genes were transcribed (Figure 2A,B) most intensively late in the night or early in the morning. As the very first components of the Ca^2+^ signalling pathway, they might have important roles in maintaining the Ca^2+^ fluctuation in plant cells. It has been demonstrated that the [Ca^2+^]_cyt._ oscillates with a circadian rhythm in plant cells [57], and this oscillation is highly influenced by the photoperiod and light intensity as well [58].

In this study, we demonstrated a wide range of analyses of the gene expression patterns of Ca^2+^ signalling-related genes. The circadian oscillation of the [Ca^2+^]_cyt_ is well known, but the complex regulatory mechanisms behind it are not well understood yet. It has been proposed [47] that this oscillation is not directly regulated by the circadian clock components themselves, but rather by the circadian modulation of those genes whose function is to modify—directly or indirectly—the activity of the Ca^2+^ channels. On the other hand, phospholipases play crucial roles in the formation of such essential secondary messengers as PA and IP_3_ or Ca^2+^ [3]. Here, we clearly show that the *HvPLD (put.)* gene was driven by the light/dark cycles (Figure 3A), thus suggesting another regulatory layer to explain the rhythmic Ca^2+^ fluctuations.

In our study the rhythmicity of some genes encoding CBPs were also demonstrated. We found that the *HvCBL2*, *HvCaM.2* and *HvCDPK12* genes are also expressed rhythmically during light/dark cycles (Figure 3B–D). These results could indicate a connection between the cytosolic [Ca^2+^] oscillation and the rhythmic transcription of CBPs.

Very few reports are available on the transcriptional profile of circadian clock-controlled *CBP*s. Among them there is one showing that rice *CaM1-1* can sense both the period and the amplitude of the circadian oscillation of cytosolic Ca^2+^ concentration during heat stress [59].

In this study, we observed low level expression in the transcript patterns for all the analysed calcium-dependent elements under constant white light conditions, even for those that had daily oscillation during light/dark cycles. In our experiment, the *HvPLC (pred.)* and the *HvCaM.1* genes (Figure S1A,B) showed neither circadian oscillation nor light-dependence at all. The expression patterns of the *HvPLC.1 (put.)* and *HvCAMTA3 (pred.)* genes (Figure S2A,B) showed that these genes were influenced by the FR light, which may play a role in the fine-tuned responses to the changes in the spectral composition of ambient light.

It is also interesting to note that the two studied *CaM* genes showed different expression patterns under the same conditions. *HvCaM.1* showed no light-response and no circadian pattern, whereas *HvCaM.2* was expressed rhythmically and showed light-dependence and a much higher expression than *HvCaM.1*. This kind of functional polymorphism might be a key element in the fine tuning of a cellular response. Whether the different regulation of these genes is based on the different *cis*-regulatory elements is a question to be answered.

### 3.3. HvCBF Genes

It is well known that a rapid, intensive cytosolic [Ca^2+^] elevation happens in the plant cells upon exposure to low temperature [14] and also that the excess of Ca^2+^ ions is originating mainly from extracellular or vacuolar stores [60]. It has been published that the *CaM-binding transcriptional activator 3* (*AtCAMTA3*) in *Arabidopsis* indeed has a role in the cold-induced expression of *AtCBF1* and *AtCBF2* genes, and also that the double *camta* mutant plants are impaired in their freezing tolerance [8], which suggests a potential link between the calcium-dependent components and the CBF regulon as well. However, it has also been proven that not all the *CBF* genes were regulated through the Ca^2+^-dependent pathway. The specific pharmacological inhibition of PLC or PLD, or the usage of Ca^2+^ blockers reduced the expression of the cold-inducible *HvCBF9* and *HvCBF14* genes from the HvCBF4-subgroup, whereas the cold-inducible *HvCBF12* or the non-cold-inducible *HvCBF6* genes from the HvCBF3-subgroup were not reduced at all in barley variety Nure [61].

The CBF transcription factors are one of the most intensively studied elements in the freezing tolerance mechanisms in *Arabidopsis* and in cereals too. It has already been described that, without cold induction, the expression of several *TaCBF* genes appear 8–14 h after dawn and also that the highest level of their expression does not match the coolest period of the day but with the daily temperature decrease during sunset, before the forthcoming chilly night [62]. On the other hand, the expression of the HvCBF4-subgroup members appeared within 1–4 h after cold induction, with the highest transcript values between 4 and 8 h at 2 °C. In addition, some of the HvCBF3-subgroup members were expressed under this condition but with a lower expression level relative to the HvCBF4-subgroup [20].

Here we found that among the HvCBF3-subgroup members only the *HvCBF3* and *HvCBF6* genes (Figure 4) were expressed at 22 °C, while all the tested HvCBF4-subgroup genes (Figure 5) were transcribed. It has already been published that the *TaCBF2*, *TaCBF9* and *TaCBF14* genes are expressed at a higher level in the winter varieties than in the spring wheat varieties [31]. An analysis [63] revealed that the *HvCBF2* and *HvCBF4* genes are encoded in higher number in the winter barley varieties Nure and Dicktoo than in the spring barley varieties Tremois and Morex. Furthermore, it was hypothesised that this increase in the copy number is responsible for the higher level of frost resistance in the winter barley genotypes [64]. It was found that the *HvCBF9* and *HvCBF14* genes localised under the *Fr-H2* locus have a major role in freezing tolerance. Moreover, based on gene expression results, it has also been concluded that the HvCBF4-subgroup contributes to enhanced frost tolerance more efficiently than the HvCBF3-subgroup in barley [65].

The differences in the HvCBF-subgroups have already been revealed at molecular level as well. It has been postulated that the HvCBF1- and HvCBF3-subgroup proteins are able to bind to the CRT motif in their targeted genes both at low and warm temperatures, whereas the HvCBF4-subgroup members are able to bind only under low temperature conditions. This temperature-dependent binding capacity may differentiate the *CBF* genes that are involved in freezing tolerance from those that are involved in other stress conditions such as drought [20]. The inductive temperature, which is low enough to up-regulate the *CBF* genes that contribute to cold acclimation in cereals, is in correlation with the level of the freezing tolerance: the activation temperature of the extreme cold tolerant wheat variety Norstar is 5.4 °C, higher than that of the slightly frost-tolerant wheat variety Manitou [66]. Furthermore, the acclimation period is shorter in the frost tolerant genotypes than in the susceptible ones [23].

In our experiment, we found that neither *HvCBF3* nor *HvCBF6* genes (HvCBF3-subgroup members) had a circadian rhythm, but all the analysed genes from the HvCBF4-subgroup (cold responsive) were controlled by the circadian clock. The connection between the oscillator and the CBF regulation has been proved at molecular level, since the expression of the *CBF* genes is directly regulated by the binding of the clock-component CCA1 to the promoter region of the *CBF* genes that induce freezing tolerance [67,68], thus suggesting that *CCA1* (and *LHY*) genes are required for proper *CBF* induction. Therefore, it can be assumed that the circadian clock plays a role in freezing tolerance by timing the cold-inducibility of the CBF regulon [33]. On the other hand, the expression of genes from the CBF4-subgroup would seem uncoupled, at least under constant light, from the expression of *CCA1* in this study.

In this study, we found that the supplemental FR light causes earlier transcription in HvCBF4-subgroup members. Moreover, we also described that the expression peaks were frequently increased compared to the gene expression level found in the white light-illuminated samples. The adequate cellular response for a change in the inner our outer environment requires the fine tuning of the signalling network. The regulation of transcription factors, one of the main regulators of gene expressions, is influenced by many input ways; the interconnected signalling pathways. It has been described first in *Arabidopsis* [43] that beside the quantity, the quality of light is also a modifier for the expression of *CBF* transcription factors. The low R/FR ratio not only increased the *CBF*s and their target *Cor15a* expression level through the phytochrome perceptional pathway, but also increased frost tolerance. In our recent work [36], the effect of low R/FR ratio on *CBF14* gene expression, which is one of the most effective ones in the *CBF* pool, was proven in barley as well. We found that a low R/FR ratio enhances the expression of *PHYA;* meanwhile it increases the *CBF14* expression and frost tolerance as well. In the current work we also prove the modifying effect of light spectra on barley *CBF*s. Like in *Arabidopsis* [43], we also found that the low R/FR ratio increased the expression level of the *CBF* genes; however, unlike in *Arabidopsis*, where this spectral modification had no or hardly any effect on the rhythmicity of *CBF* gene expression, we also noticed that the periodicity was indeed influenced. All the genes belonging to the HvCBF4-subgroup showed an earlier expression maximum—compared to the samples illuminated by unmodified spectral light. This earlier and higher level of expression we found here—even under a non-inductive circumstance—might have an adaptive role in low temperature stress tolerance. Changes in the light spectra during a day influences plant physiology in many ways. It is known that R/FR ratio drops in dusk, preceding the forthcoming cooler night. Thus, the perception of changes in the daily spectral composition leads to an earlier and elevated level of cold tolerance through the phytochrome—CBF regulon pathway.

## 4. Materials and Methods

### 4.1. Plant Material and Growth Conditions

A cold tolerant, winter habit barley (*Hordeum vulgare* spp. *vulgare*) variety, Nure was used in this experiment. After germination in Petri dishes (1 day at 25 °C and 3 days at 4 °C) 150 seedlings were planted into wooden boxes. The growing medium was a 2:1:1 (*v*/*v*/*v*) mixture of soil, sand and humus. The plantlets were grown in plant growth chambers (Conviron PGV36; Controlled Environments Ltd.; Winnipeg, MB, Canada) with 12 h photoperiods, at 20 °C/17 °C (day/night), with 70–75% RH, at 250 µmol m^−2^·s^−1^ light intensity under cool white fluorescent tubes (Sylvania 215 W F96 T12). After one week of preliminary growth, the temperature was raised to a steady 22 °C, but the other environmental parameters—except the light—were not changed any more. After 5 days at 22 °C, half of the plants started to receive supplemental FR light treatment (see below) in developmental phase Z13 according to the Zadoks scale [69], whereas the remaining plants were further treated with fluorescent white light only, and considered as controls (growth conditions are illustrated in Figure S3). Plants were inside the same growth chamber with non-reflective white separators between treatments to prevent light contamination.

### 4.2. Light Treatment

The supplemental FR (735 nm) light was added to the fluorescent white light by 3 W high-power LED panels (Shenzhen Justar Electronic Technology Co., Ltd., Guangdong, China). Based on [43], we used the lowest R/FR ratio, which provided a stable 0.5 R/FR ratio in our plant growth chamber. The light intensity was unchanged during the whole experiment. With the exception of the FR light supplement, the treated and the control plants were subjected to the same environmental factors during the whole experiment.

### 4.3. Sampling for Gene Expression Studies

After one week of supplemental FR light treatment, leaf samples were collected every 4 h during the following 4 days. On the 1st and 2nd days, a 12 h photoperiod was applied, while, on the 3rd and 4th days, the experiments were run under constant light conditions. The very first sample was collected immediately after the light switched on, i.e., at the beginning of the 8th day. At every sampling time leaves were collected from the FR light-treated plants and the control plants as well; each sample consisted of a mixture of leaves from three independent plants, which were pooled and homogenized for RNA isolation.

### 4.4. Gene Expression Studies

The total RNA from the leaves was isolated by Direct-zol^TM^ RNA MiniPrep kit (Zymo Research Corp., Irvine, CA, USA) according to the manufacturer’s instructions. The RNA quantification was carried out by a NanoDrop 2000 Spectrophotometer (Thermo Fisher Scientific Inc., Wilmington, MA, USA). cDNA syntheses were made using the M-MLV Reverse Transcriptase and oligo(dT)_15_ primer (Promega Corporation, Madison, WI, USA) according to the manufacturer’s protocol. The gene expression levels were determined with the CFX96 Touch^TM^ Real-Time PCR Detection System (Bio-Rad Hungary Ltd., Budapest, Hungary) using the KAPA SYBR^®^ FAST, Master Mix (2×), Universal qPCR Kit (Kapa Biosystems, Inc., Wilmington, MA, USA).

Some genes were obtained from previously published papers, or were retrieved from the NCBI nucleotide database (based on the gene name and species), while others were searched through NCBI BLAST alignment (Table S1). However, in several cases only putative genes or cds for a predicted protein sequence were found. For each gene, we selected the nucleotide sequences with the best Query cover, E-value and Ident value for barley sequences (Table S1). The primers (Table S1) were designed with the NCBI—Primer Design Tool (National Center for Biotechnology Information, Bethesda, MD, USA) and the Oligo Analyzer (developer: Teemu Kuulasmaa, version 1.0.3) software, or the sequences were taken from the literature [70,71,72]. In every case a melt curve was determined to confirm the amplification of a single gene product. The relative expression levels were calculated by the Δ*C*_t_ method [73] using *cyclophilin* as a reference gene for normalisation, since its transcript level was not affected by any treatments. Thus, the expression values are comparable across the genes and the treatments as well.

In this study, each data point represents a single sample, which is a mixture of leaves collected from three independent plants, and which was homogenized for RNA isolation, then used for cDNA syntheses. This cDNA sample was used for qRT-PCR, and three amplifications (from the same cDNA sample) were considered as technical replicates.

## 5. Conclusions

Those cold inducible *HvCBF* genes, which are known to play a role in the enhancement of frost tolerance and expressed even at a higher temperature (*HvCBF2A*, *HvCBF4B*, *HvCBF9* and *HvCBF14*), were found being controlled by the circadian clock in barley. The observed transcription pattern of the HvCBF4-subgroup members (i.e., the ones in which the peaks in their expression occur in the afternoon) suggests that they may contribute to the avoidance of those damages that might happen due to the unfavourable conditions (lower temperatures, freezing) during the nights, since the CBF-regulated, stress-protecting effector genes are switched on in advance. The effect of the supplemental FR light resulted in earlier gene-induction and higher peaks in many cases, which may have implications in enhancement to cold conditions. The analysed phospholipid signalling members, i.e., *PITP* and *PI4K*, which indirectly contribute to the cytosolic [Ca^2+^] changes, show a circadian rhythm as well. The supplemental FR light resulted in earlier *PI4K* gene induction, just like in the case of the HvCBF4-subgroup.

**Figure 6:**
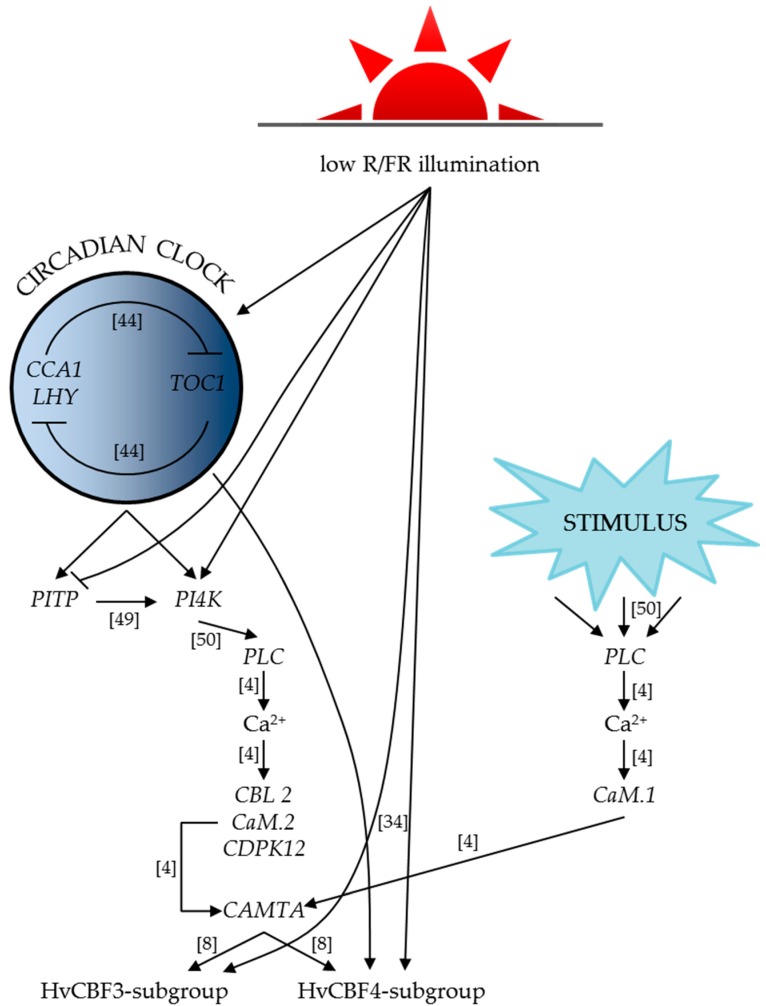
Graphical summary of our results based on the gene expression analyses. The abbreviations (R/FR, *CCA1*, *LHY*, *PITP*, *PI4K*, *PLC*, *CBL2*, *CaM.2*, *CDPK12* and *CAMTA*) and the references in the brackets are detailed in the text. Lines ending with arrowheads represent induction, while lines with blunt ends mean inhibition.

Based on our gene expression results, we revealed the connections between the circadian clock, the phospholipid signalling pathway, the calcium signalling elements and the *HvCBF*s genes, and constructed a graphical summary to illustrate these relations (Figure 6). Our findings suggest that the effect of the genes involved in the development of low temperature tolerance is highly influenced by the spectral composition of the light. These results show that stress tolerance may be enhanced by the modulation of light, which promises possible practical benefits and economic advantages as well, for example, in indoor plant growing systems.

## Supplementary Materials

Supplementary materials can be found at www.mdpi.com/1422-0067/18/8/1828/s1.
