# Supplementary material for: Circadian and Light Regulated Expression of CBFs and their Upstream Signalling Genes in Barley
Source: Int J Mol Sci. 2017 Aug 22;18(8):1828. doi: 10.3390/ijms18081828 (PMC5578212; doi:10.3390/ijms18081828)
Supplement: Supplementary file 1 [file ijms-18-01828-s001.zip › Supplemental Figures (Round 3).pdf]

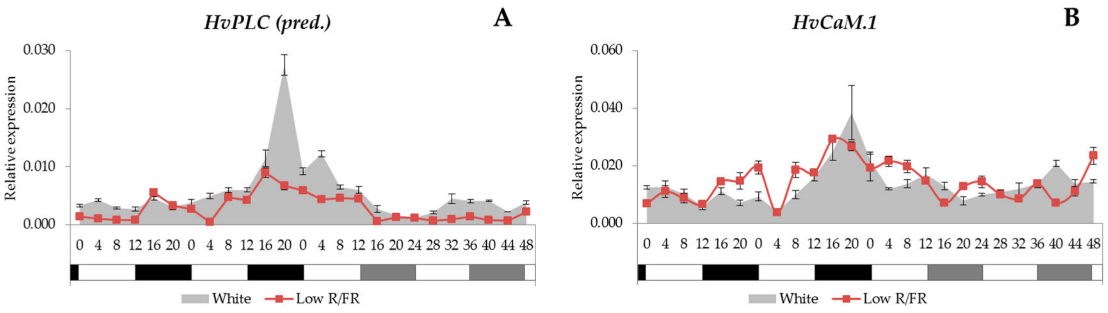

Supplemental Figure S1. Expression patterns of *HvPLC (pred.)* and *HvCaM.1* (panels A, B, respectively) with white light and low R/FR illumination. Conditions are the same as in Figure 1.

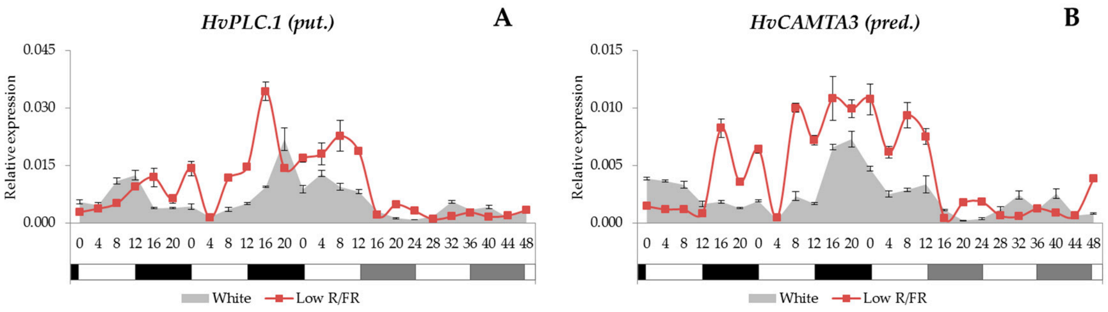

Supplemental Figure S2. Gene expression patterns of the *HvPLC.1 (put.)* and *HvCAMTA3 (pred.)* (panels A, B, respectively) with white light and with supplemental FR in the spectra. Conditions are the same as in Figure 1.

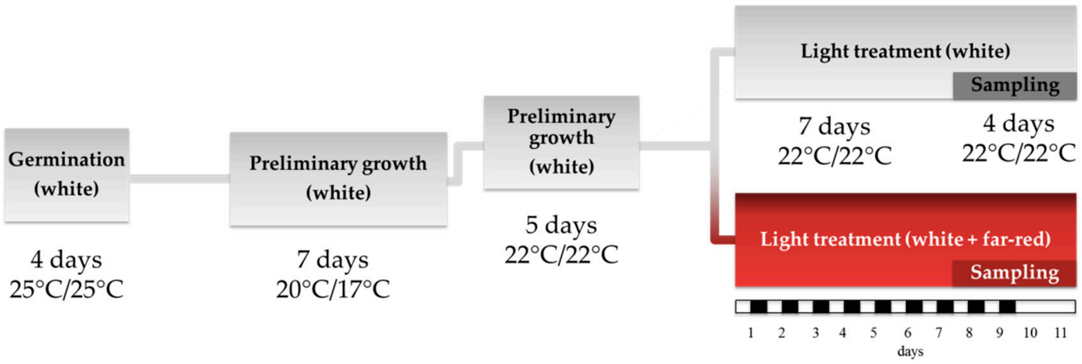

Supplemental Figure S3. Graphical representation of growth conditions and sample collection during the whole experiment. Details are in the manuscript.
